# Sex differences in global metabolomic profiles of COVID-19 patients

**DOI:** 10.1038/s41419-022-04861-2

**Published:** 2022-05-14

**Authors:** Rocio Diaz Escarcega, Pedram Honarpisheh, Gabriela Delevati Colpo, Hilda W. Ahnstedt, Lucy Couture, Shivanki Juneja, Glenda Torres, Guadalupe J. Ortiz, James Sollome, Natalie Tabor, Bhanu P. Ganesh, H. Alex Choi, Fudong Liu, Louise D. McCullough, Andrey S. Tsvetkov

**Affiliations:** 1 Department of Neurology, the University of Texas McGovern Medical School at Houston, Houston, TX USA; 2 The University of Texas Graduate School of Biomedical Sciences, Houston, TX USA; 3 Department of Neurosurgery, the University of Texas McGovern Medical School at Houston, Houston, TX USA; 4 Metabolon, Inc., Morrisville, NC USA; 5 UTHealth Consortium on Aging, the University of Texas McGovern Medical School, Houston, TX USA

**Keywords:** Metabolomics, Immunological disorders

## Abstract

Coronavirus disease (COVID-19), caused by SARS-CoV-2, leads to symptoms ranging from asymptomatic disease to death. Although males are more susceptible to severe symptoms and higher mortality due to COVID-19, patient sex has rarely been examined. Sex-associated metabolic changes may implicate novel biomarkers and therapeutic targets to treat COVID-19. Here, using serum samples, we performed global metabolomic analyses of uninfected and SARS-CoV-2-positive male and female patients with severe COVID-19. Key metabolic pathways that demonstrated robust sex differences in COVID-19 groups, but not in controls, involved lipid metabolism, pentose pathway, bile acid metabolism, and microbiome-related metabolism of aromatic amino acids, including tryptophan and tyrosine. Unsupervised statistical analysis showed a profound sexual dimorphism in correlations between patient-specific clinical parameters and their global metabolic profiles. Identification of sex-specific metabolic changes in severe COVID-19 patients is an important knowledge source for researchers striving for development of potential sex-associated biomarkers and druggable targets for COVID-19 patients.

## Introduction

Coronavirus disease (COVID-19) caused by SARS-CoV-2 leads to a wide spectrum of symptoms, ranging from asymptomatic disease to death [1–4]. A few vaccines are effective against SARS-CoV-2, and a few therapeutics (e.g., remdesivir and steroids) are available [4]. However, new approaches to manage COVID-19-related complications are urgently needed.

Although women and men have similar infection rates, men have higher risks of hospitalization, ICU care, and mortality secondary to infection [5–7]. Data on sex differences in long-term outcomes (“COVID-19 long haulers”) point to higher COVID-19-related mental health complications in women [8]. However, the underlying mechanisms are poorly understood. Although men may have more comorbidities and engage in more risky behaviors, sex-associated differences in COVID-19 severity persist even after controlling for these [5].

Interestingly, most COVID-19 studies ignored sex as a variable [9]. SARS-CoV-2 enters the cell by binding the angiotensin-converting enzyme (ACE) 2 receptor [10]. Some studies found sex-associated differences in ACE2 expression, but others did not [11, 12]. Expression of the ACE2 receptor and TMPRSS2 (the cellular serine protease for priming) and innate and adaptive immunity are regulated by sex hormones and may contribute to sex-associated differences in COVID-19 [11, 13]. However, advanced age is a risk factor for COVID-19 morbidity and mortality and makes the role of sex hormones less likely.

Sex differences in the immune response may explain sex-dependent responses to COVID-19 [14–20]. Levels of several pro-inflammatory chemokines and cytokines are higher in affected males [16]. The T-cell response is more robust in females than male patients, and poor male T-cell responses correlate with COVID-19 progression [16]. The immune response is modulated by key metabolic pathways, which are regulated by the host, microbiome, and metabolic cross-talk.

Here, we used mass spectrometry and machine-learning algorithms for untargeted metabolomic analyses of uninfected and severe COVID-19 male and postmenopausal female patient serum samples (20 individuals per group, appropriately powered, matched for severity). The most robust sexually dimorphic metabolic pathways in COVID-19 groups, but not in controls, involved lipid metabolism, pentose pathway, bile acid metabolism, and microbiome-related metabolism of aromatic amino acids, including tryptophan (e.g., serotonin) and tyrosine. Our findings indicate that SARS-CoV-2 triggers profound host and microbiome metabolic shifts with key sex differences that might be used to develop sex-specific diagnostics and therapies for COVID-19 patients.

## Results

### Selection of COVID-19 patient samples

Houston, Texas, experienced an initial surge in COVID-19 cases in March-April and a resurgence in June-August, 2020 [21]. We analyzed serum samples from 20 male and 20 female patients with severe COVID-19 [22], and 20 male and 20 female controls. Patients with severe COVID-19 were matched to controls, based on age, race and comorbidities by the nearest-neighbor propensity score-matching method (the R package ‘matchit’) (Supplemental Table 1 and see Methods).

### Global metabolomic profiling showed differences in male and female patients and control subjects

**Fig. 1:**
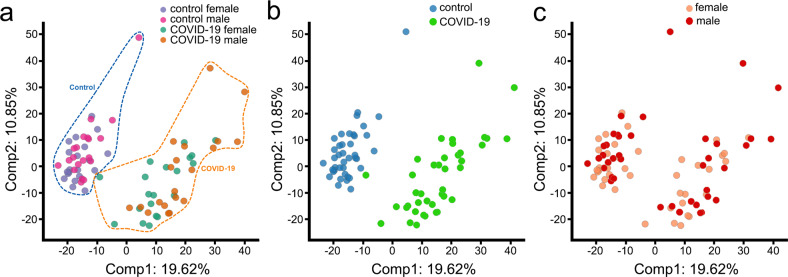
Global metabolomic profiling of male and female COVID-19 patients and control individuals. Principal component analysis (PCA) is used to define metabolomic profiles. **a** PCA of metabolomic data for 20 male and female COVID-19 patients and control subjects. Each dot represents an individual subject. PCA demonstrates segregation of serum samples based on a group. The percentage values refer to the percentage of total variance associated with each component. Note how disease status (control versus severe COVID-19) and sex contribute in Note how disease status (control versus COVID-19) and sex contribute in different ways to sample clustering (depicted with dashed lines). Purple: control female subjects, magenta: control male subjects, aquamarine: female COVID-19 patients, orange: male COVID-19 patients. **b** PCA demonstrates segregation of serum samples, based on disease status (control versus severe COVID-19). Blue: control male and female subjects, green: male and female COVID-19 patients. **c** PCA shows segregation of serum samples based on sex. Salmon: female control subjects and COVID-19 patients, red: male control subjects and COVID-19 patients.

Serum samples were loaded in an equivalent manner across the analytical platforms (Metabolon). In the serum sample dataset, 1516 named and unnamed biochemicals were detected by principal components analysis (PCA). PCA employs orthogonal transformation to highlight similarities and differences and to detect patterns within the dataset. Samples with similar metabolic profiles tend to group together, and those with different profiles segregate. Control and COVID-19 samples clearly separated (Fig. 1a). Metabolic profiles differed dramatically in relation to the SARS-CoV-2 infection state but less by patient sex (Fig. 1b, c).

**Fig. 2:**
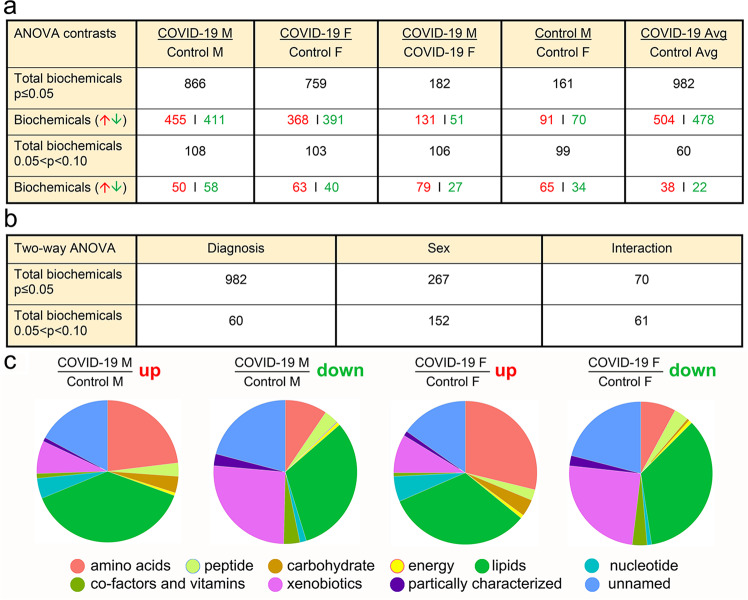
Metabolite summary and significantly altered biochemicals. **a** The dataset comprises 1516 biochemicals, 1214 compounds of known identity (named biochemicals), and 302 compounds of unknown structural identity (unnamed biochemicals). ANOVA contrasts were used to identify biochemicals that differed significantly between experimental groups. A summary of the numbers of biochemicals that achieved statistical significance (*p* ≤ 0.05), as well as those approaching significance (0.05 < *p* < 0.10) is shown. **b** Analysis by two-way ANOVA identified biochemicals exhibiting significant interaction and main effects for experimental parameters of COVID-19 and sex. **c** A summary of biochemical families that achieved statistical significance (*p* ≤ 0.05). Ten families of metabolic biochemicals were identified: amino acids, peptides, carbohydrates, energy, lipids including primary and secondary bile acid metabolites, nucleotides, co-factors and vitamins, xenobiotics, partially characterized and unnamed biochemicals.

Next, ANOVA contrasts were used to examine 866 biochemicals that differed in males and 759 in females. Of these, 182 biochemicals differed in male and female patients, and 161 differed in male and female controls. Some approached significance (0.05 < *p* < 0.10) (Fig. 2a). Thus, some biochemicals exhibited interactions with COVID-19 diagnosis and sex (Fig. 2b).

We grouped the biochemicals according to their super pathways and types. Biochemicals fell into several metabolic pathways (Fig. 2c). Numerous xenobiotics, including drugs, environmental pollutants, and agricultural chemicals, and partially characterized and unnamed biochemicals, were discovered.

### Random forest metabolite classifications

**Fig. 3:**
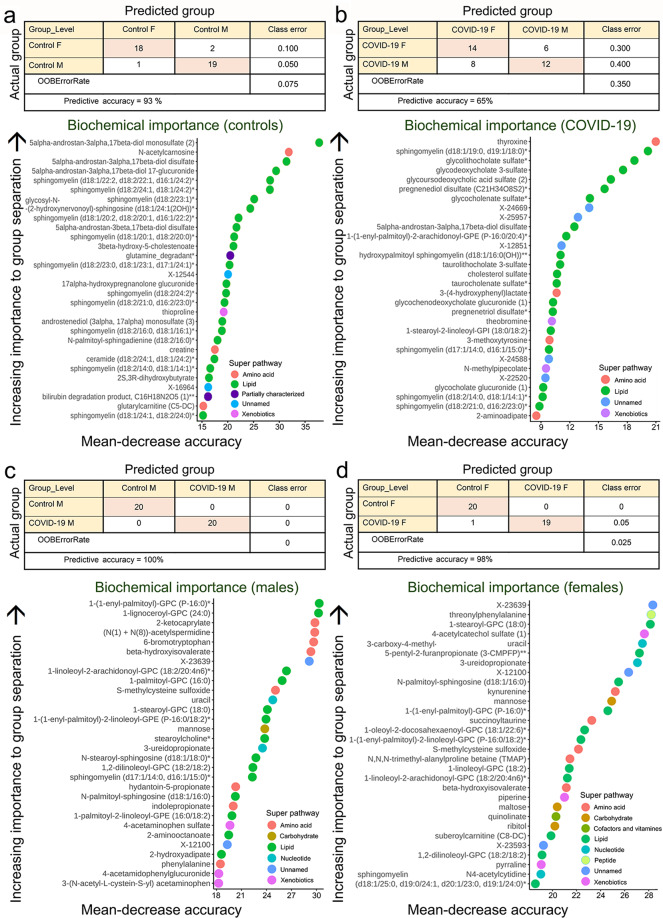
Random forest analysis of metabolic serum datasets. Random forest analysis bins individual samples into groups, based on their metabolite similarities and variances. Colored dots represent metabolite super pathway families. Salmon: amino acids, green: lipids, dark purple: partially characterized molecules, blue: unnamed biochemicals, magenta: xenobiotics, brown: carbohydrates, turquoise: nucleotides, olive: co-factors and vitamins, yellow: peptides. **a** The biochemical profiles predict the control sample group with a predictive accuracy of 93% (control male and female individuals) (top). Random forest analysis shows the 30 most important metabolites in the control group (male and female subjects) (bottom). **b** The biochemical profiles predict the COVID-19 sample group with a predictive accuracy of 65% (male and female COVID-19 patients) (top). Random forest analysis demonstrates top 30 ranking biochemicals of importance, based on male COVID-19 and female COVID-19 group separation in serum samples (bottom). **c** The biochemical profiles are highly successful in predicting the male groups correctly with 100% accuracy (control and COVID-19 samples) (top). Random forest analysis demonstrates the top 30 ranking biochemicals of importance, based on control male and COVID-19 male group separation in serum samples (bottom). **d** The biochemical profiles are highly successful in predicting the female groups correctly with 98% accuracy (control and COVID-19 samples) (top). Random forest analysis demonstrates the 30 most important metabolites in the female group (control and COVID-19 subjects) (bottom).

Random forest analysis bins individual samples into groups, based on metabolite similarities and variances, which may identify biomarkers of interest. Biochemical profiles predicted the controls and patients with accuracies of 93% and 65%, respectively (Fig. 3a, b) and predicted the sample male and female groups correctly with accuracies of 100% and 98% (Fig. 3c, d), respectively. In control groups, the 30-top ranking biochemicals showed differences in lipid and amino acid metabolism. In patients, they differed in lipid and amino acid metabolism and unnamed biochemicals; however, these metabolites differed in the controls. In males, the 30-top biochemicals indicated key differences in lipid and amino acid metabolism. In females, the 30-top biochemicals suggested key differences in lipid and amino acid metabolism. The major changes in both male and female sera, which may serve as biomarkers, were lipid and amino acid metabolites.

### Lipid metabolism in patients

**Fig. 4:**
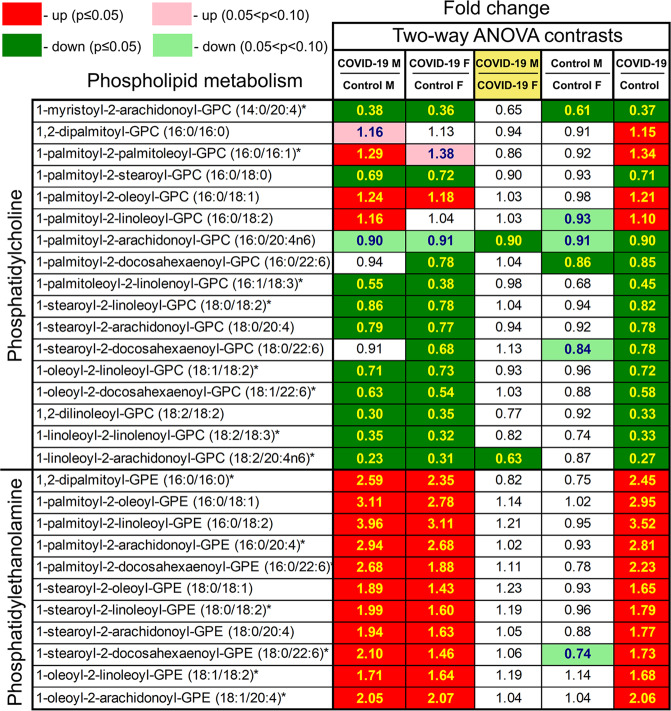
Differences in phospholipid metabolism between control subjects and COVID-19 patients. Red and green cells indicate *p* ≤ 0.05 (red indicates the fold-change values are significantly higher for that comparison; green values significantly lower). Light red and light green shaded cells indicate 0.05 < *p* < 0.10 (light red indicates the fold-change values trend higher for that comparison; light green values trend lower). Note that phosphatidylcholine metabolites are in general lower in male and female COVID-19 samples than controls, and phosphatidylethanolamine metabolites are higher in male and female COVID-19 samples than controls.

Phospholipid phosphatidylcholine (PC) and phosphatidylethanolamine (PE) are membrane phospholipids. Patients had fewer PC metabolites (e.g., 1-palmitoyl-2-stearoyl-GPC (16:0/18:0) and 1,2-dilinoleoyl-GPC (18:2/18:2)) than controls with minor sex-associated differences (Fig. 4). Patients had more PE metabolites than controls with no sex-dependent differences. Several long-chain fatty acids were higher in COVID-19 patients than controls (e.g., pentadecanoate (15:0), palmitoleate (16:1n7), and docosapentaenoate (22:5n3)) (Supplemental Fig. 1). These differences might indicate an altered state of fatty-acid utilization in SARS-CoV-2-positive subjects.

In most tissues, beta-oxidation is regulated by coenzyme A and mitochondrial uptake of fatty acids by the carnitine shuttle. Initially, fatty acids are activated by long-chain acyl-CoA synthetase to form acyl-CoA (e.g., palmitoyl-CoA). The acyl group is transferred to the hydroxyl group of carnitine to form acylcarnitine (e.g., palmitoylcarnitine) in the outer membrane of the mitochondria. Acylcarnitine can then enter the mitochondrial matrix to be utilized for beta-oxidation and ATP generation. Levels of several dicarboxylate and hydroxy-carnitine-conjugated fatty acids (e.g., adipoylcarnitine, suberoylcarnitine, and 3-hydroxydecanoylcarnitine) are higher in the COVID-19 samples than controls (Supplemental Data 1 and 2). An indicator of an excess of acetyl-CoA is an elevation of the ketone body 3-hydroxybutyrate, which is also elevated in COVID-19 patients (Supplemental Figure 2). Since fatty-acid beta-oxidation occurs in mitochondria, SARS-CoV-2 infection might alter mitochondrial function in response to virus-induced energy demand. Our data suggest a significant difference in fatty-acid metabolism (Supplemental Fig. 3 and Supplemental Data 1 and 2).

**Fig. 5:**
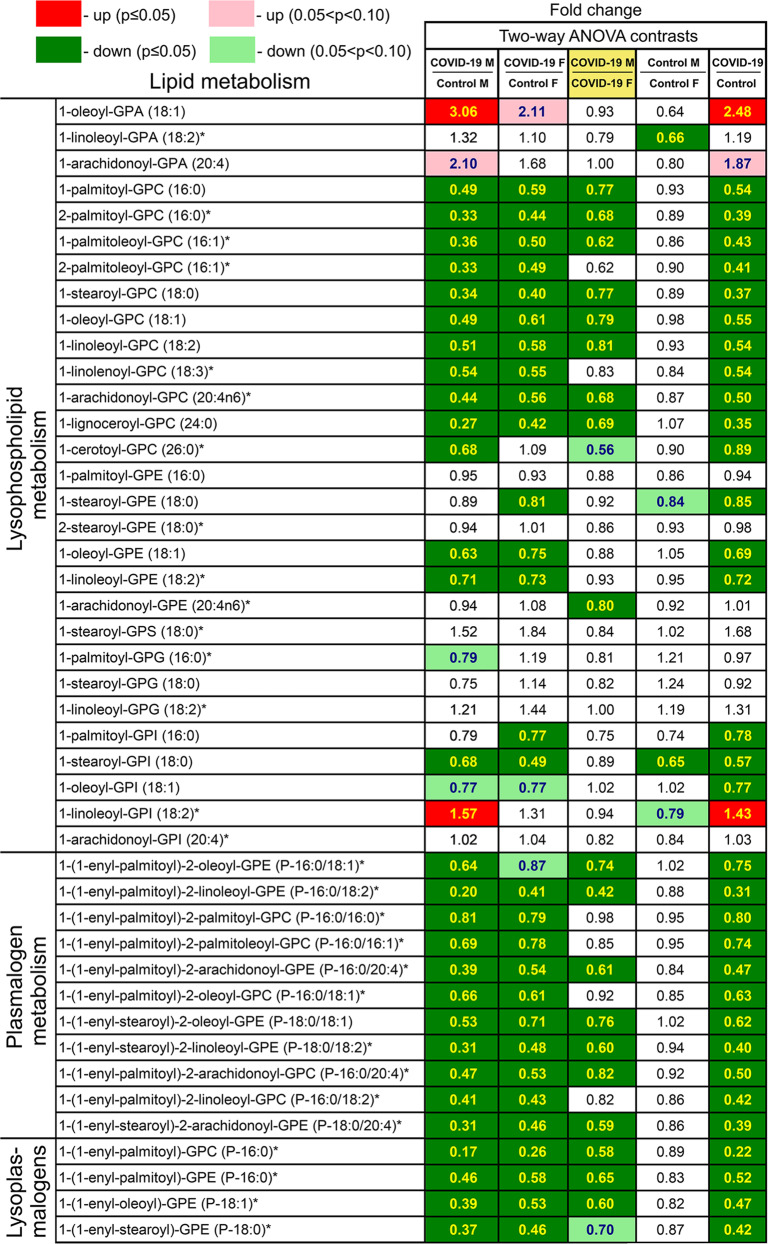
Differences in metabolism of lysophospholipids, plasmalogens, and lysoplasmalogens between control subjects and COVID-19 patients. Red and green cells indicate *p* ≤ 0.05 (red indicates the fold-change values are significantly higher for that comparison; green values significantly lower). Light red and light green shaded cells indicate 0.05 < *p* < 0.10 (light red indicates the fold-change values trend higher for that comparison; light green values trend lower). Note that most metabolites considerably reduced in COVID-19 patients with a sex association. These metabolites are lower in male patients than in female patients.

Metabolites of lysophospholipids, plasmalogens, and lysoplasmalogens were considerably reduced in patients and reduced more in male patients (Fig. 5). These lipid metabolites exhibit one of the most dramatic sex-dependent differences.

Other phospholipid metabolites (e.g., trimethylamine N-oxide, glycerophosphoserine) were altered in SARS-CoV-2-positive groups with sex-associated differences (Supplemental Data 1 and 2). Since viruses target lipid synthesis and signaling within the host to produce lipids for viral envelopes, differences in phospholipid metabolites may indicate active viral replication.

### Bile acid metabolism is altered in a sex-associated manner

**Fig. 6:**
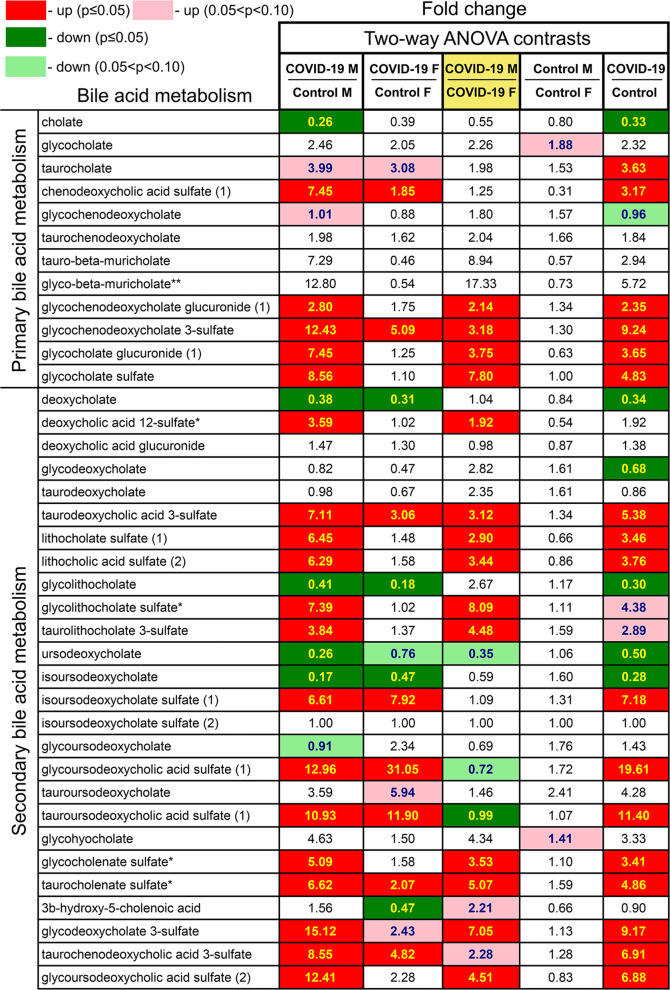
Differences in metabolism of primary and secondary bile acids between control subjects and COVID-19 patients. Red and green cells indicate *p* ≤ 0.05 (red indicates the fold-change values are significantly higher for that comparison; green values significantly lower). Light red and light green shaded cells indicate 0.05 < *p* < 0.10 (light red indicates the fold-change values trend higher for that comparison; light green values trend lower). Note that there is a sex association for many metabolites.

**Fig. 7:**
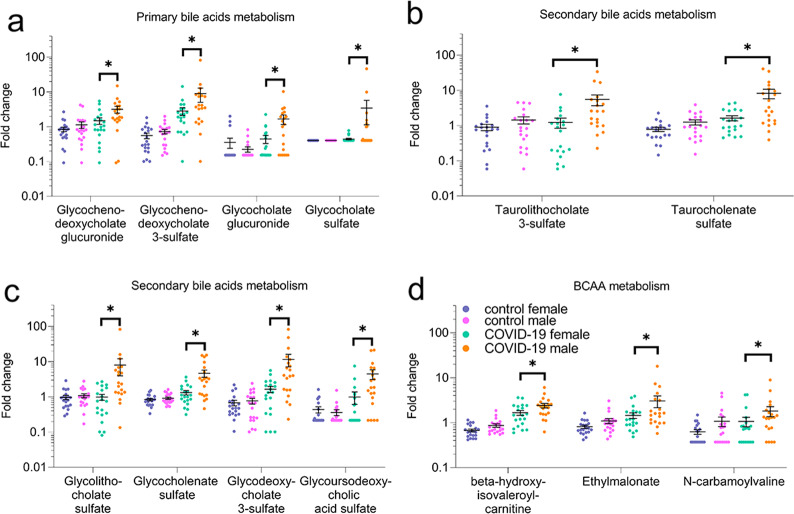
Sex differences in primary and secondary bile acids metabolism pathways and in valine, isoleucine, leucine (BCAA) metabolism in COVID-19 groups, not present in control groups. **a** Primary bile acid metabolism and **b**, **c** secondary bile acid metabolism pathways. **d** Branched-chain amino acids, valine, isoleucine, leucine metabolism, *p ≤ 0.05 (two-way ANOVA).

Bile acids regulate intestinal nutrient absorption, secretion of lipids, toxic metabolites, xenobiotics, hepatic lipid, glucose, and energy homeostasis. We identified strong signatures of primary and secondary bile acid metabolism often in a sex-dependent manner (Figs. 6 and 7). Some metabolites have no sex-associated differences in controls, but do in patients. For example, glycocholate sulfate was not altered in female patients but strongly elevated in male patients, indicating possible liver dysfunction in males.

### Amino acid metabolism in patients

Amino acid metabolism was one of the most altered pathways in patients (Figs. 2 and 3, Supplemental Fig. 2, Supplemental Data 1 and 2). Metabolites of branched-chain amino acids were profoundly changed. They are degraded into carbon skeletons that may enter gluconeogenesis or fatty-acid synthesis or energy pathways (TCA cycle). Most branched-chain amino acid metabolites were elevated with some sex-associated differences (Fig. 7d, Supplemental Fig. 2). For example, beta-hydroxyisovaleroylcarnitine, ethylmalonate, and N-carbamoylvaline did not differ between male and female control individuals, but all three metabolites were higher in male COVID-19 patients (Fig. 7d). The accumulation of the downstream metabolites may indicate increased branched-chain amino acid degradation and/or a reduced incorporation into the TCA cycle mediated by altered mitochondrial function.

Other amino acid metabolites were also elevated. For example, lysine, polyamine, and methionine metabolites were elevated in patients with minor sex differences (Supplemental Data 1 and 2). Metabolites of glycine, serine, threonine, alanine, aspartate, glutamate, histidine, and methionine were mostly elevated in patients with minor sex differences. Some sex differences in amino acid metabolites existed with COVID-19 males having more of them. Thyroxine, a tyrosine metabolite, was a rare exception; it was unaltered in female patients and reduced in males. Most amino acid metabolites were altered in patients, and most were elevated in those with severe COVID-19.

### Altered carbohydrate and energy metabolism in COVID-19 patients

**Fig. 8:**
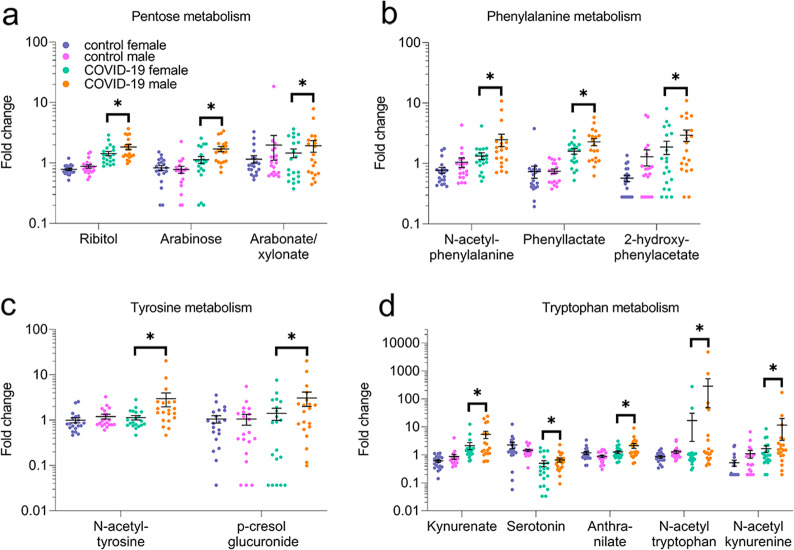
Sex differences in pentose metabolites and microbiome related metabolites in COVID-19 groups, not present in control groups. **a** Pentose, **b** phenylalanine, **c** tyrosine and, **d** tryptophan metabolism pathways. *p ≤ 0.05 (two-way ANOVA).

Glucose is metabolized in the glycolytic pathway, in which glucose is converted to glucose-6-phosphate and then to pyruvate and lactate. Pyruvate enters the TCA cycle and alters energy states. Several pentose-related metabolites (e.g., ribitol, arabinose, and arabitol/xylitol) exhibited a strong sex association in COVID-19 patients only, and were higher in male than female patients (Fig. 8a, Supplemental Fig. 3). Glucose, pyruvate, and lactate levels were greater in the patients than controls, with pyruvate trending higher in male than female patients. Energy-related metabolites citrate and isocitrate were lower in the patients than controls, whereas alpha-ketoglutarate was higher in the patients than in controls.

### Microbiome-related metabolism in patients

Microbial action within the gut facilitates metabolism of aromatic amino acids (e.g., phenylalanine, tyrosine, and tryptophan), secondary bile acids, and benzoates [23, 24]. Levels of phenylalanine and tyrosine were higher, but tryptophan was lower in patients than controls (Supplemental Fig. 4). Several phenylalanine metabolites (e.g., N-acetyl-phenylalanine, phenyllactate, and 2-hydroxy- phenyllactate) exhibited a strong sex dependency and were higher male than female patients (Fig. 8b, Supplemental Figure 4). In the tyrosine metabolism pathway, N-acetyl-tyrosine and p-cresol-glucuronide were considerably higher in males with COVID-19 with no sex-associated differences in controls (Fig. 8c). N-acetyl-tryptophan was 17-fold higher in male patients with no sex differences in controls. N-Acetyl-kynurenine was sevenfold higher in male patients with no sex-associated differences in controls. Kynurenic acid was 2.5-fold higher in males with COVID-19 with no sex-associated differences in controls (Fig. 8d, Supplemental Data 1 and 2). Quinolinate, a neurotoxic downstream product of the kynurenine pathway, was increased in COVID-19 samples with a potential sex-association (0.05 < p < 0.1). Serotonin levels were lower in patients than controls and higher in male than female patients (Fig. 8d, Supplemental Fig. 4). Other microbiome-related metabolites (Supplemental Fig. 4) and benzoate metabolites were altered in the COVID-19 groups, compared to controls (Supplemental Data 1 and 2). Infection alters the state of microbiome, and sex-associated differences in aromatic amino acids and secondary bile acids (Fig. 6) may result from infection or infection and differences in diet and lifestyle.

### Higher oxidative stress and proinflammatory metabolites in patients

Oxidative stress in viral infections may result in lipid peroxidation and protein and DNA/RNA oxidation. Some oxidative stress-related metabolites were different in the SARS-CoV-2-positive group (Supplemental Data 1 and 2). Eicosanoid 12-hydroxyeicosatetraenoic acid, an inflammatory biochemical, was higher in patients of both sexes. Pro-inflammatory 13-hydroxyoctadecadienoic acid and 9-hydroxyoctadecadienoic acid were elevated in male and female patients. A long-chain polyunsaturated fatty acid, docosapentaenoate (22:5n3), was increased in patients of both sexes (Supplemental Fig. 1).

### Purine and pyrimidine metabolism in patients

Several classes of purine and pyrimidine metabolites were higher in patients than controls. For example, xanthosine, N1-methyladenosine, orotate, and 3-methylcytidine were higher in patients than controls (Supplemental Data 1 and 2). Exceptions exist: 5-methyluridine, 2′-deoxyuridine, and dihydroorotate were reduced in male and female patients (Supplemental Data 1 and 2). Several metabolites exhibited strong sex-association and were higher in male than female patients. Thus, increased purine and pyrimidine metabolites in COVID-19 likely indicate cellular and mitochondrial damage.

### Metabolism of co-factors and vitamins

We detected 40 co-factors and vitamins in serum samples (Supplemental Data 1 and 2). Co-factors and vitamins are in general decreased in patients. However, 2-O-methylascorbic acid was greater in male and female patients. L-Urobilin was dramatically increased in male patients and could be a marker of severe COVID-19 in male patients. Most co-factor and vitamin metabolites, however, were reduced in COVID-19 patients. With higher levels of L-urobilin, heme levels were lower in male than female patients. Vitamins A and B5 (pantothenate) and retinal were reduced in both patients. Thus, most co-factors and vitamins and their metabolites were reduced in COVID-19 patients with a few exceptions, such as L-urobilin and heme, that exhibited a strong sex-association (Supplemental Data 1 and 2).

### Steroids and their metabolites in COVID-19

Of 46 steroids, about one-third did not differ in any groups (Supplemental Data 1 and 2). Another third differed in non-infected male and female controls. Several steroids were dramatically increased in patients with a strong sex dependency. For example, pregnenetriol disulfate, 5alpha-pregnan-diol disulfate, and tetrahydrocortisol sulfate were increased in patients and even higher in males. Estrone sulfate was greater in COVID-19 male than female patients (with no difference between control males and females). A few androgenic steroids, including dehydroepiandrosterone sulfate, 16a-hydroxy DHEA 3-sulfate, androsterone glucuronide, epiandrosterone sulfate, and androstenediol (3alpha, 17alpha) monosulfate, were reduced in both male and female patients (Supplemental Data 1 and 2). Thus, profound changes in steroids in patients may result in oxidative stress, inflammation, cognitive impairment, depression, and other pathologies.

### Partially characterized biochemicals, unnamed molecules, and xenobiotics

Partially structurally characterized and unnamed biochemicals were identified by their chromatographic and mass spectral signatures. Some differ in controls and patients with a minor sex association (Supplemental Data 1 and 2). Finally, some xenobiotics (e.g., drugs, environmental pollutants, and agricultural chemicals) were lower in patients than controls, likely due to lower exposure and continuous excretion of these pollutants.

### Unsupervised investigation of correlation between patient-specific clinical parameters and global metabolic profile reveals significant sexual dimorphism

To identify potential biomarkers of hospitalized patients’ responses to SARS-CoV-2, we performed high-throughput, unsupervised correlation analysis of the medical record data from individual patient inpatient course and past medical history (approximately 50 clinical parameters) with their global metabolic profile (1,516 named and unnamed metabolites). Patient age, BMI, days in ICU, days on mechanical ventilator, comorbidities, mortality due to COVID-19, and more were included in our analysis (Supplemental Table 2 for complete list). Differences in metabolites strongly correlated with clinical parameters between sex-matched controls and patients (Supplemental Fig. 5). Importantly, differences in metabolic profiles strongly correlated with clinical parameters of male and female patients. Our results (Supplemental Table 3) are promising starting points for future searches for targetable serum metabolites in managing COVID-19.

## Discussion

We compared metabolomic profiles of serum samples from male and postmenopausal female severe COVID-19 patients and uninfected individuals. We restricted our cohorts to severe cases to minimize the known male bias in severity. The greatest sex-aggregated changes were in lipid and amino acid metabolites and metabolism of carbohydrates, energy, nucleotides, steroids, and the microbiome-related pathways. Oxidative stress-related metabolites were elevated, reflecting elevated production of reactive oxygen species. Proinflammatory metabolites were considerably upregulated in COVID-19 patients. Key pathways strongly sex-associated in COVID-19 patients involved lipid metabolism, pentose pathway, bile acid metabolism, and microbiome-related metabolism of aromatic amino acids, including tryptophan and tyrosine (Supplemental Fig. 6). Unfortunately, a disappointing majority of COVID-19 studies ignored sex as a variable [9, 25–30]. As COVID-19 is a sex-associated disease, a major novelty of our study is the inclusion of sex as a biological variable in severe COVID-19 patients.

Our findings clearly establish COVID-19 as a metabolically sex-associated disease (Supplemental Fig. 6). Our study was appropriately powered with 20 individuals per group with both sexes. Importantly, our dataset contains 1214 named and 302 unnamed biochemicals. With Metabolon’s global precision metabolomics LC-MS platform, we discovered that 866 biochemicals were altered in male patients with 455 biochemicals upregulated and 411 downregulated. In female patients, 759 compounds were altered with 368 biochemicals upregulated and 391 downregulated. Male and female COVID-19 samples differed: 182 biochemicals differed between the two groups. Metabolomic differences manifested in the serum of patients compared to control subjects and revealed differences in male and female patients.

Why is COVID-19 a sex-associated disease? A recent study controlled for potential comorbidities, such as hypertension, cardiovascular disease, and diabetes and risky behaviors in males and still found sex-associated differences in COVID-19 severity [5]. Importantly, male patients exhibit higher levels of several pro-inflammatory chemokines and cytokines, such as IL-8, IL-18 and CCL5 [16]. Female COVID-19 patients have more robust T-cell activation than male patients, and the association between poor T-cell responses and worse disease outcomes only occurs in male patients [16]. Metabolic signatures of blood biomarkers linked to low-grade inflammation and cardiometabolic diseases collected a decade before the pandemic correlate with susceptibility to COVID-19 [31]. Sex hormones regulate the immune response to infections and also regulate expression of the ACE2 receptor and TMPRSS2, which are responsible for viral entry and priming, respectively [11]. Sex hormones may contribute to the observed sex differences in COVID-19 outcomes; however, age-related susceptibility to severe COVID-19 makes a dominant role of sex hormones less likely. Properly designed studies, including peri- and postmenopausal cohorts with or without hormone replacement therapies, with a well-documented timeline with respect to the presence or absence of a prolonged period of gonadal senescence, can shed some light on any potential roles of sex hormones in response to COVID-19 [32].

In our study, all female patients were postmenopausal (Supplemental Table 1). Intriguingly, we observed changes in levels of estrogenic and androgenic steroids with some sex-associated differences. For example, estrone sulfate, an abundant circulating estrogen in non-pregnant women and men secreted by the ovaries and adipose tissues, were profoundly higher in COVID-19 male patients than female patients (with no difference between control males and females). A number of androgenic steroids were dramatically decreased in male and female patients. These steroid changes could lead to oxidative stress, inflammation, and cognitive impairment.

Sex differences in the acute and chronic immune response to the SARS-CoV-2 infection may also contribute to the sex-dependent mechanisms in COVID-19. We discovered that metabolites of lysophospholipids, plasmalogens, and lysoplasmalogens were reduced more in male patients. Although these lipid metabolites may differentially regulate the immune system in male and female patients, they regulate myriad non-immune signaling pathways [33, 34]. Intriguingly, platelets are important in thrombotic complications of COVID-19 [35, 36], with female patients being less predisposed to thromboembolism [37]. Known sex differences in the coagulation cascade may contribute to short and long-term outcomes of COVID-19 [38, 39].

Primary and secondary bile acid metabolism was also altered in patients in a sex-dependent manner (Fig. 7). As bile acids and their metabolites are critical for nutrient and drug absorption and distribution [40], they may contribute to sex differences in response to therapies for COVID-19. Our study may provide a foundation for improved disease diagnosis and interventions with the awareness that male and female COVID-19 patients have different metabolic profiles. COVID-19 patients exhibited altered microbiome-related metabolism, with a few robust sex differences in metabolism of key aromatic amino acids and secondary bile acids (Figs. 7 and 8). These changes could result from COVID-19-induced changes in the microbiome [41, 42] or differences in patient diet and lifestyle. Notably, all female patients and 18 of 20 males were receiving nasogastric tube feeding. This strengthens our conclusions by reducing short-term dietary variabilities, a challenge in clinical studies.

Plasmalogens and lysoplasmalogens were reduced in COVID-19 patients and even more so in male patients (Fig. 5, Supplemental Fig. 6). Plasmalogens are abundant membrane glycerophospholipids, and lysoplasmalogens are generated from plasmalogens by hydrolysis that releases polyunsaturated fatty acids from glycerol sn-2 carbon [43]. In many diseases, including age-associated neurodegeneration, heart and metabolic diseases, plasmalogens decrease, leading to inflammation and cellular stress [43]. Exogenous plasmalogens may be a therapy for ameliorating disease phenotypes [44, 45]. Importantly, there is a strong association between plasmalogen levels and patient sex. Lower plasmalogens in male patients may partially explain why men are more susceptible to the disease.

We found that several metabolites of the kynurenine pathway (KP) showed a significant increase in the sex-aggregated COVID-19 groups but not in control groups. In previous studies, 17 metabolites (e.g., amino acids tryptophan, proline, and glutamine) were associated with the disease, and the amount of the tryptophan metabolite kynurenic acid highly correlated with age, inflammation, and disease outcome in male patients only [46]. Importantly, we found a difference by sex when comparing tryptophan and KP metabolites in male and female patients. Kynurenic acid, a KP metabolite, may regulate sex-specific immune responses in COVID-19 by activating the aryl hydrocarbon receptor (AHR) [46]. AHR is an environmental sensor that integrates microbiome-derived and host-derived metabolic signals to regulate the innate and adaptive immune responses [47, 48]. AHR activation may be a strategy by coronaviruses to evade antiviral immunity, and pharmacological inhibition of AHR has been proposed as a COVID-19 therapy [48–50].

Another clinically important microbiome-related change involved serotonin (Fig. 8, Supplemental Fig. 4). Tryptophan metabolism and the brain-gut-microbiome axis are critical for the serotonergic system normal functions, and the possible consequences of imbalanced tryptophan metabolism are pain, anxiety, cognitive impairments, and depression [51]. Interestingly, women who have or had COVID-19 are more likely to develop psychiatric symptoms and are lonelier than men [8]. Serotonin levels were lower in sex-aggregated COVID-19 patients than controls. Female patients had less serotonin than male patients, which may reflect dysfunctional tryptophan and KP metabolism from COVID-19-induced changes in the microbiome. Altered tryptophan metabolism and serotonin may induce COVID-19-related post-traumatic stress disorder that disproportionally affects women [8].

In summary, our global metabolomics profile showed a metabolic shift in COVID-19 patients and sex-associated pathways. Lipids, bile acids, and aromatic amino acid metabolites in severe COVID-19 patients depended on sex, and an interplay of host-regulated and microbiome-regulated metabolic pathways appears to be a major underlying mechanism of sex differences in short- and long-term responses to COVID-19.

## Methods

### Patient sample selection

Serum samples were from male and female severe COVID-19 patients and uninfected individuals, which were collected pre-pandemic with the exception of two male uninfected controls collected during the pandemic. Informed consent was obtained from all subjects. 20 male and 20 female patients with severe COVID-19 were matched to male and female controls, based on age, race and number of comorbidities by the nearest neighbor propensity score matching method with the R package ‘matchit’. The mean of N comorbidities in non-COVID-19 versus COVID-19 was 2.4 (SD = 2.04) versus 2.3 (SD = 2.03) within female groups, and 2.45 (SD = 1.47) versus 2.40 (SD = 1.47) within male groups, with *p* values well above 0.05. The mean BMI of patients in the severe COVID-19 cohort was 33.19 ± 9.1 for females and 35.79 ± 11.08 for males. COVID-19 samples were collected 24–72 h after admission. Female patients were on a feeding tube for 18.7 ± 16.7 days, and male patients were on a feeding tube for 18.9 ± 11.5 days. Severe COVID-19 illness was classified as the following: individuals who have SpO2 < 94% on room air at sea level, a ratio of arterial partial pressure of oxygen to fraction of inspired oxygen (PaO2/FiO2) < 300 mm Hg, respiratory frequency >30 breaths/min, or lung infiltrates >50%.

### Sample accessioning and preparation

Serum samples were collected under the approved IRBs (“BioRepository of neurological diseases and sex-specific immune responses to SARS-CoV-2 and chronic neurologic symptoms”, HSC-MS-17-0452; “Neutrophil-DEspR+ in COVID”, HSC-MS-20-0780), inventoried, assigned with a unique identifier, and stored at −80 °C. This identifier was used to track all sample handling, experiments, and results. Serum samples were prepared using the automated MicroLab STAR® system from Hamilton Company. Proteins were precipitated with methanol under vigorous shaking for 2 min and followed by centrifugation. The resulting extract was divided into five fractions: two for analysis by two separate reverse phase (RP)/UPLC-MS/MS methods with positive ion mode electrospray ionization (ESI), one for analysis by RP/UPLC-MS/MS with negative ion mode ESI, one for analysis by HILIC/UPLC-MS/MS with negative ion mode ESI, and one sample was reserved for backup.

### Ultrahigh-performance liquid chromatography-tandem mass spectroscopy (UPLC-MS/MS)

All methods used a Waters ACQUITY UPLC and a Thermo Scientific Q-Exactive high-resolution/accurate MS interfaced with a heated electrospray ionization (HESI-II) source and Orbitrap mass analyzer operated at 35,000 mass resolution. The sample extract was dried and then dissolved in solvents compatible to each of the four methods. Each solvent contained a series of standards to ensure injection and chromatographic consistency. One aliquot was analyzed using acidic positive ion conditions, chromatographically optimized for more hydrophilic compounds. A C18 column (Waters UPLC BEH C18-2.1 × 100 mm, 1.7 µm) was used, as well as water and methanol, containing 0.05% perfluoropentanoic acid (PFPA) and 0.1% formic acid (FA). Another aliquot was analyzed using acidic positive ion conditions; however, it was chromatographically optimized for more hydrophobic compounds. The extract was eluted from the same C18 column using methanol, acetonitrile, water, 0.05% PFPA and 0.01% FA. Another aliquot was analyzed using basic negative ion optimized conditions using a separate dedicated C18 column. The extracts were eluted from the column using methanol and water, however, with 6.5 mM ammonium bicarbonate, pH 8. The fourth aliquot was analyzed via negative ionization, after elution from an HILIC column (Waters UPLC BEH Amide 2.1 × 150 mm, 1.7 µm) using a gradient of water and acetonitrile with 10 mM ammonium formate, pH 10.8. The MS analysis alternated between MS and data-dependent MSn scans using dynamic exclusion.

### Data extraction and compound identification

Raw data were extracted, peak-identified, and processed using Metabolon’s hardware and software. Compounds were identified by comparison to library data of purified standards or recurrent unknown entities. The library contains the retention time/index (RI), mass to charge ratio (m/z), and chromatographic data (including MS/MS spectral data) on all molecules present in the library. More than 3300 commercially available purified standard compounds have been acquired and registered. Additional mass spectral data have been created for structurally unnamed biochemicals, which were identified by their chromatographic and mass spectral signatures.

### Statistical methods

Standard statistical analyses were performed in Jupyter Notebook or R or JMP.

### Random forest

This supervised classification technique is based on an ensemble of decision trees [52]. For a given decision tree, a random subset of the data with identifying true class information is selected to build the tree (“training set”), and then, the remaining data, the “out-of-bag” (OOB) variables, are passed down the tree to obtain a class prediction for each sample. This process is repeated thousands of times to produce the forest. The final classification of each sample is determined by computing the class prediction frequency (“votes”) for the OOB variables over the whole forest. The prediction accuracy is an unbiased estimate of how well one can predict sample class in a new data set. R was used for the random forest method.

### Principal components analysis

Principal components analysis is an unsupervised analysis that reduces the dimension of the data. Each principal component is a linear combination of every metabolite, and the principal components are uncorrelated. The number of principal components is equal to the number of observations. The first principal component is computed by determining the coefficients of the metabolites that maximizes the variance of the linear combination. The second component finds the coefficients that maximize the variance with the condition that the second component is orthogonal to the first. The third component is orthogonal to the first two components and so on. The total variance is defined as the sum of the variances of the predicted values of each component (the variance is the square of the standard deviation), and for each component, the proportion of the total variance is computed.

### Unsupervised correlation analysis of clinical outcomes with global metabolomics data

Fifty clinical outcome parameters, including death due to COVID-19, days in critical care, days on invasive mechanical ventilator, coagulation parameters (e.g., INR, D-dimer, and aPTT), white blood cell counts, patient history of serious illnesses (e.g., cancer, diabetes mellitus, stroke, or congestive heart failure), and several other parameters from the patient electronic medical records were used to perform unsupervised Pearson correlation analysis. Sex-disaggregated data tables were constructed, and each of the clinical parameters was separately analyzed with all 1516 named and unnamed metabolites detected in our global metabolomics profiling dataset. Batch-normalized imputed values were used for performing the correlation analyses. The source code was written in MATLAB 2021a and will be made available on GitHub for open access upon publication. Volcano plots were generated in GraphPad Prism.

## Supplementary information

Supplemental figure and table legends

Supp Table 1

Supp Table 2

Supp Table 3

Supp Figure 1

Supp Figure 2

Supp Figure 3

Supp Figure 4

Supp Figure 5

Supp Figure 6

Supplemental Data 1

Supplemental Data 2

Conformation from all co-authors to agree to add a new co-author.

## Data Availability

Metabolomics data is available in article supplementary material.
