# Supplementary material for: Sex differences in global metabolomic profiles of COVID-19 patients
Source: Cell Death Dis. 2022 May 14;13(5):461. doi: 10.1038/s41419-022-04861-2 (PMC9106988; doi:10.1038/s41419-022-04861-2)
Supplement: Supplementary file 1 — Supplemental figure and table legends [file 41419_2022_4861_MOESM1_ESM.docx]

**Supplemental figure and table legends**

**Supplemental Table 1. Clinical characteristics of COVID-19 patients**. Serum samples from 20 male and 20 female control individuals and 20 male and 20 female COVID-19 patients were used for analyses by ultrahigh-performance liquid chromatography-tandem mass spectroscopy.

**Supplemental Table 2. The list of clinical parameters extracted from patients’ electronic medical records for performing the unsupervised correlation analysis**. The dataset comprises 58 clinical parameters and the binary codes (0=no, 1=yes) used for performing the unsupervised Pearson correlation analysis.

**Supplemental Table 3. Tabulated results for the metabolites that were strongly correlated with each clinical parameter.**The dataset comprises the list of metabolic profiles correlated with clinical parameters. Threshold for -log(q-value) was chosen to be greater than or equal to 5.0.

**Supplemental Figure 1. Elevated free fatty acids with SARS-CoV-2 infection**. Red and green cells indicate p≤0.05 (red indicates the fold-change values are significantly higher for that comparison; green values significantly lower). Light red and light green shaded cells indicate 0.05<p<0.10 (light red indicates the fold-change values trend higher for that comparison; light green values trend lower). Note that most metabolites are increased in COVID-19 patients with no sex-association.

**Supplemental Figure 2. Differences in metabolism of branched-chain amino acids, valine, isoleucine, and leucine** **in control subjects and COVID-19 patients**. Red and green cells indicate p≤0.05 (red indicates the fold-change values are significantly higher for that comparison; green values significantly lower). Light red and light green shaded cells indicate 0.05<p<0.10 (light red indicates the fold-change values trend higher for that comparison; light green values trend lower). Note that most metabolites are increased in COVID-19 patients with a few examples of a dependency on patient sex.

**Supplemental Figure 3. Altered carbohydrate and energy metabolism in COVID-19 patients**. Red and green cells indicate p≤0.05 (red indicates the fold-change values are significantly higher for that comparison; green values significantly lower). Light red and light green shaded cells indicate 0.05<p<0.10 (light red indicates the fold-change values trend higher for that comparison; light green values trend lower).

**Supplemental Figure 4. Differences in microbiome related metabolism between control subjects and COVID-19 patients.** Red and green cells indicate p≤0.05 (red indicates the fold-change values are significantly higher for that comparison; green values significantly lower). Light red and light green shaded cells indicate 0.05<p<0.10 (light red indicates the fold-change values trend higher for that comparison; light green values trend lower). Note that the aromatic amino acids phenylalanine and tyrosine are both significantly higher, and tryptophan is significantly lower in the COVID-19 group than controls. Several aromatic amino acid metabolites (e.g., N-acetylphenylalanine, 3-(4-hydroxyphenyl)lactate, and kynurenate) are significantly higher in the COVID-19 group than controls, and significantly higher in the COVID-19 male group than the COVID-19 female group. Also note that serotonin is significantly lower in the COVID-19 groups than controls, but lower in the COVID-19 female group than the COVID male group (see Discussion).

**Supplemental Figure 5. Unsupervised investigation of correlation between patient-specific clinical parameters and global metabolic profile reveals significant sexual dimorphism**.

Volcano plots of Pearson correlation coefficient and -log(adj p-value) for correlation analysis between all clinical parameters (listed in Supplementary Table 4) and metabolomics profiles of **(a)** control males, **(b)** control females, **(c)** COVID-19 males, and **(d)** COVID-19 females depicts significant sex differences.

**Supplemental Figure 6. A summary of the most profound sex-associated differences in severe COVID-19 male and female patients**. Key sex-associated metabolites with profound changes involved lipid metabolism, pentose pathway, bile acid metabolism, and microbiome-related metabolism of aromatic amino acids, including tryptophan and tyrosine. Red (up) and green (down) numbers indicate the ratio of fold changes between male and female patients with severe COVID-19.

**Supplemental Data 1**. The statistical heat map associated with the statistical analyses of the raw data.

**Supplemental Data 2**. The raw data generated from 20 male and female COVID-19 patients and control subjects using ultrahigh-performance liquid chromatography-tandem mass spectroscopy.
