# Supplementary material for: Sex differences in global metabolomic profiles of COVID-19 patients
Source: Cell Death Dis. 2022 May 14;13(5):461. doi: 10.1038/s41419-022-04861-2 (PMC9106988; doi:10.1038/s41419-022-04861-2)
Supplement: Supplementary file 2 — Supp Table 1 [file 41419_2022_4861_MOESM2_ESM.docx]

| \| Table 1. Clinical information of controls and COVID-19 patients \| \| \| \| \| \| --- \| --- \| --- \| --- \| --- \| \| Variable \| Control (N=40) \| \| Severe COVID-19 (40) \| \| \| Sex no. (%) \| \| \| \| \| \| Female \| 20 (50.0) \| \| 20 (50.0) \| \| \| Male \| 20 (50.0) \| \| 20 (50.0) \| \| \| Age (year) \| Female \| Male \| Female \| Male \| \| Mean (SD) \| 62.05 ± 12.98 \| 61.95 ± 16.90 \| 60.50 ± 18.73 \| 58.85 ±18.59 \| \| Media (IQR) \| 64 (72-50) \| 63.5 (72.75-47.5) \| 64.5 (72-51) \| 57.5 (69.75-49.75) \| \| Range \| 41-89 \| 24-96 \| 27-95 \| 24-101 \| \| Race no. (%) \| \| \| \| \| \| Black \| 6 (30.0) \| 2 (10.0) \| 2 (10.0) \| 3 (15.0) \| \| Hispanic \| 1 (5.0) \| 5 (25.0) \| 5 (25.0) \| 9 (45.0) \| \| UTD \| 0 (0.0) \| 1 (5.0) \| 1 (5.0) \| 1 (5.0) \| \| White \| 13 (65.0) \| 12 (60.0) \| 12 (60.0) \| 7 (35.0) \| \| BMI \|  \|  \|  \|  \| \| Mean (SD) \| 28.81 ± 6.69 \| 28.89 ± 5.32 \| 33.19 ± 9.10 \| 35.79 ± 11.08 \| \| Median (IQR) \| 30.54 (34.1-22.1) \| 28.52 (29.2-27.0) \| 30.40 (35.7-28.4) \| 32.6 (43.3-29.2) \| \| Range \| 20.5-39.5 \| 29.9-41.4 \| 20.4-56.1 \| 22.7-69.3 \| \| Comorbidity no. (%) \|  \|  \|  \|  \| \| Mean (SD) \| 2.05 ± 1.70 \| 2.25 ± 1.70 \| 2.60 ± 2.21 \| 2.65 ± 1.72 \| \| Hyperlipidemia \| 11(55.0) \| 8 (40.0) \| 6 (30.0) \| 5 (25.0) \| \| Hypertension \| 10 (50.0) \| 10 (50.0) \| 11 (55.0) \| 14 (70.0) \| \| Obesity \| 5 (25.0) \| 2 (10.0) \| 8 (40.0) \| 10 (50.0) \| \| Diabetes I \| 2 (10.0) \| 3 (15.0) \| 0 (0.0) \| 2 (10.0) \| \| Diabetes II \| 0 (0.0) \| 2 (10.0) \| 9 (45.0) \| 7 (35.0) \| \| Cancer \| 7 (35.0) \| 6 (30.0) \| 1 (5.0) \| 1 (5.0) \| \| Coronary Artery Disease \| 1 (5.0) \| 4 (20.0) \| 2 (10.0) \| 4 (20.0) \| \| CHF \| 2 (10.0) \| 2 (10.0) \| 1 (5.0) \| 2 (10.0) \| \| Carotid Stenosis \| 1 (5.0) \| 1 (5.0) \| 1 (5.0) \| 0 (0.0) \| \| Atrial Fibrillation \| 2 (10.0) \| 2 (10.0) \| 4 (20.0) \| 1 (5.0) \| \| Ischemic Stroke \| 0 (0.0) \| 1 (5.0) \| 1 (5.0) \| 1 (5.0) \| \| Intracerebral Hemorrhage \| 0 (0.0) \| 1 (5.0) \| 2 (10.0) \| 1 (5.0) \| \| Stroke (type unknown) \| 0 (0.0) \| 2 (10.0) \| 2 (10.0) \| 3 (5.0) \| \| ESRD \| 0 (0.0) \| 1 (5.0) \| 2 (10.0) \| 2 (5.0) \| \| Coagulation Disorder \| 0 (0.0) \| 0 (0.0) \| 2 (10.0) \| 0 (0.0) \| \| Characteristics of COVID-19 patient / Time from onset to admission (days) \| \| \| \| \| \| Mean (SD) \|  \|  \| 7.57 ± 6.12 \| 6.85 ± 6.59 \| \| Median (IQR) \|  \|  \| 6.5 (7.75-4.25) \| 5 (7.75-3.25) \| \| Range \|  \|  \| 2-26 \| 1-27 \| \| Diagnoses no. (%) \|  \|  \|  \|  \| \| COVID-19 \|  \|  \| 20 (100) \| 19 (95.0) \| \| Other \|  \|  \| 0 (0.0) \| 1 (5.0) \| \| COVID-19 symptoms no. (%) \| \| \| \| \| \| Fever \|  \|  \| 8 (40.0) \| 7 (35.0) \| \| Cough \|  \|  \| 11 (55.0) \| 10 (50.0) \| \| Shortness of breath/difficulty breathing \|  \|  \| 19 (95.0) \| 20 (100) \| \| Diarrhea \|  \|  \| 3 (15.0) \| 2 (10.0) \| \| Chills \|  \|  \| 4 (20.0) \| 3 (15.0) \| \| Anosmia \|  \|  \| 1 (5.0) \| 1 (5.0) \| \| Ageusia \|  \|  \| 0 (0.0) \| 3 (15.0) \| \| Other \|  \|  \| 5 (25.0) \| 8 (40.0) \| \| Other COVID-19 information \|  \|  \|  \|  \| \| ICU no. (%) \|  \|  \| 19 (95.0) \| 20 (100) \| \| Mean of Days in ICU (SD) \|  \|  \| 22.85 ± 18.06 \| 21.05 ± 13.14 \| \| Invasive mechanical ventilator \|  \|  \| 19 (95.0) \| 19 (95.0) \| \| Mean of Days mechanical ventilator \|  \|  \| 19.58 ± 17.23 \| 14.30 ± 11.05 \| \| ECMO no. (%) \|  \|  \| 3 (15.0) \| 3 (15.0) \| \| Death due to COVID-19 \|  \|  \| 19 (95.0) \| 12 (60.0) \|   no. (%), number; SD, standard deviation; IQR, interquartile range; Congestive Heart Failure, CHF; End Stage Renal Disease, ESRD; intensive care unit, ICU; ECMO, Extracorporeal membrane oxygenation: UTD, unable to determine. |
| --- | --- | --- | --- | --- | --- | --- | --- | --- | --- | --- | --- | --- | --- | --- | --- | --- | --- | --- | --- | --- | --- | --- | --- | --- | --- | --- | --- | --- | --- | --- | --- | --- | --- | --- | --- | --- | --- | --- | --- | --- | --- | --- | --- | --- | --- | --- | --- | --- | --- | --- | --- | --- | --- | --- | --- | --- | --- | --- | --- | --- | --- | --- | --- | --- | --- | --- | --- | --- | --- | --- | --- | --- | --- | --- | --- | --- | --- | --- | --- | --- | --- | --- | --- | --- | --- | --- | --- | --- | --- | --- | --- | --- | --- | --- | --- | --- | --- | --- | --- | --- | --- | --- | --- | --- | --- | --- | --- | --- | --- | --- | --- | --- | --- | --- | --- | --- | --- | --- | --- | --- | --- | --- | --- | --- | --- | --- | --- | --- | --- | --- | --- | --- | --- | --- | --- | --- | --- | --- | --- | --- | --- | --- | --- | --- | --- | --- | --- | --- | --- | --- | --- | --- | --- | --- | --- | --- | --- | --- | --- | --- | --- | --- | --- | --- | --- | --- | --- | --- | --- | --- | --- | --- | --- | --- | --- | --- | --- | --- | --- | --- | --- | --- | --- | --- | --- | --- | --- | --- | --- | --- | --- | --- | --- | --- | --- | --- | --- | --- | --- | --- | --- | --- | --- | --- | --- | --- | --- | --- | --- | --- | --- | --- | --- | --- | --- | --- | --- | --- | --- | --- | --- | --- | --- | --- | --- | --- | --- | --- | --- | --- | --- | --- | --- | --- | --- | --- | --- | --- | --- | --- | --- | --- | --- | --- | --- | --- | --- | --- | --- | --- | --- | --- | --- | --- | --- | --- | --- | --- | --- | --- | --- | --- | --- | --- | --- | --- | --- | --- | --- | --- | --- | --- | --- | --- | --- | --- | --- | --- | --- | --- | --- | --- | --- | --- | --- | --- | --- | --- | --- | --- |
