# Supplementary figures and images for: Sex differences in global metabolomic profiles of COVID-19 patients
Source: Cell Death Dis. 2022 May 14;13(5):461. doi: 10.1038/s41419-022-04861-2 (PMC9106988; doi:10.1038/s41419-022-04861-2)

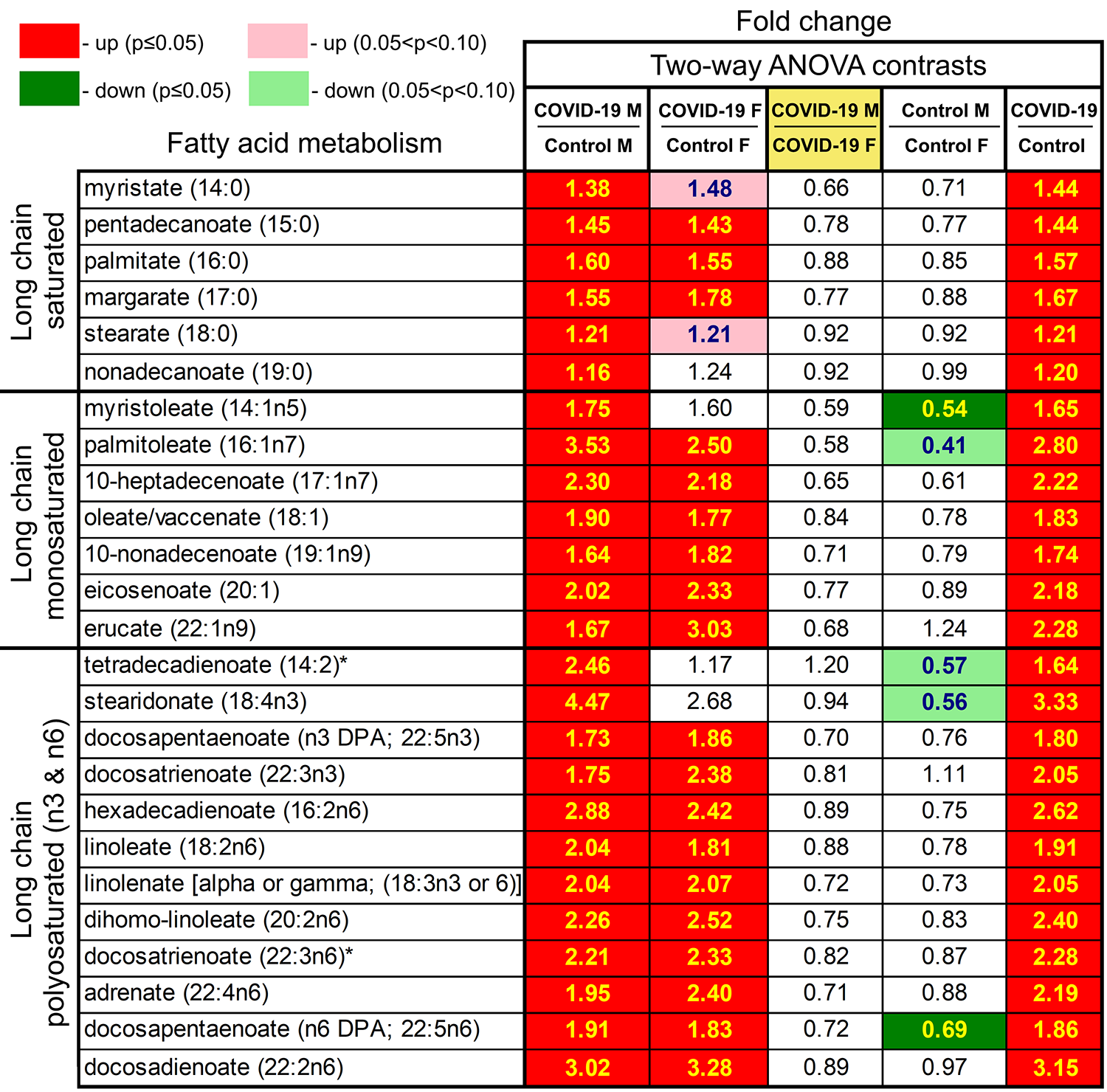

Supplement: Supplementary file 5 — Supp Figure 1 [file 41419_2022_4861_MOESM5_ESM.tif]

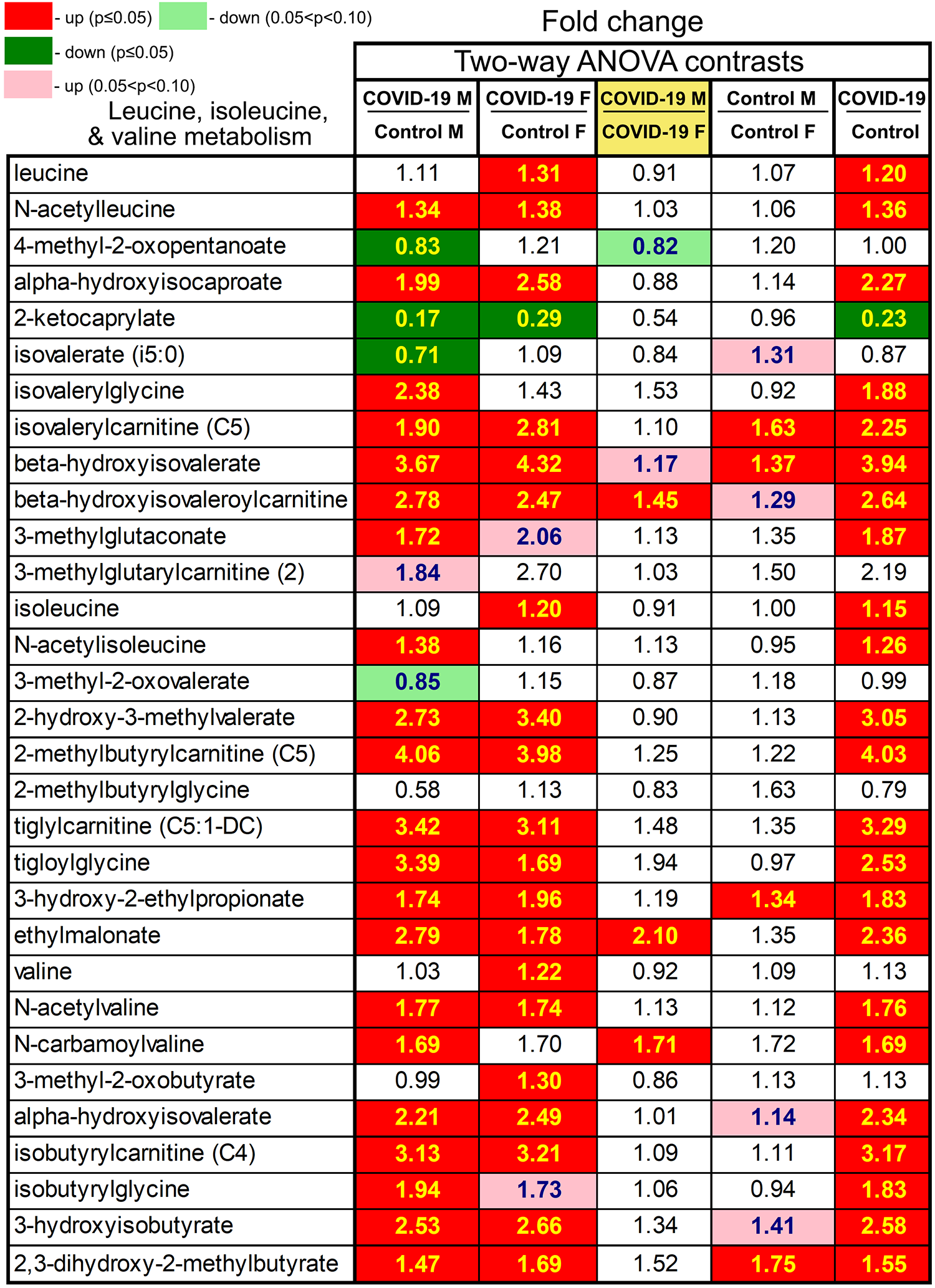

Supplement: Supplementary file 6 — Supp Figure 2 [file 41419_2022_4861_MOESM6_ESM.tif]

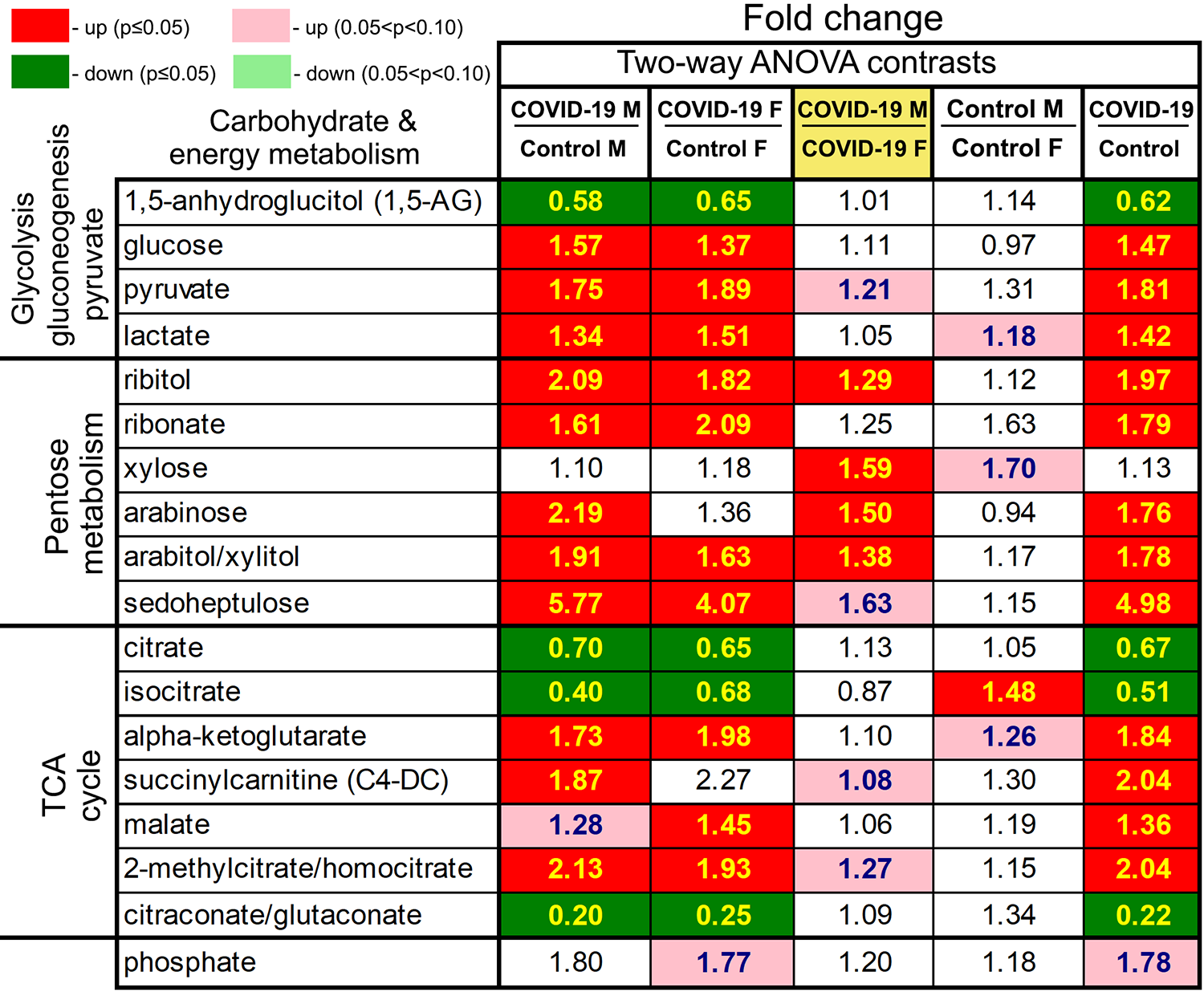

Supplement: Supplementary file 7 — Supp Figure 3 [file 41419_2022_4861_MOESM7_ESM.tif]

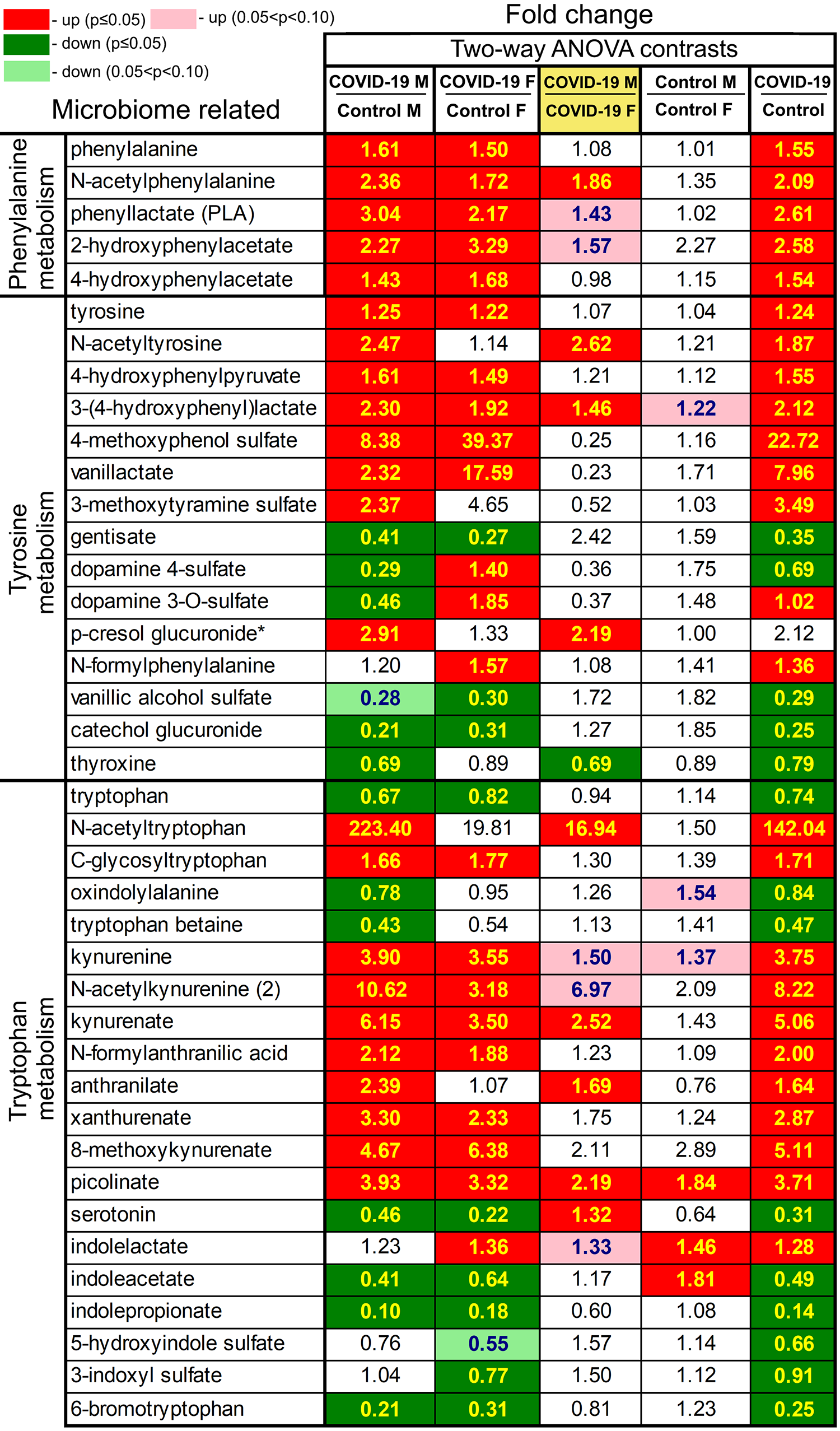

Supplement: Supplementary file 8 — Supp Figure 4 [file 41419_2022_4861_MOESM8_ESM.tif]

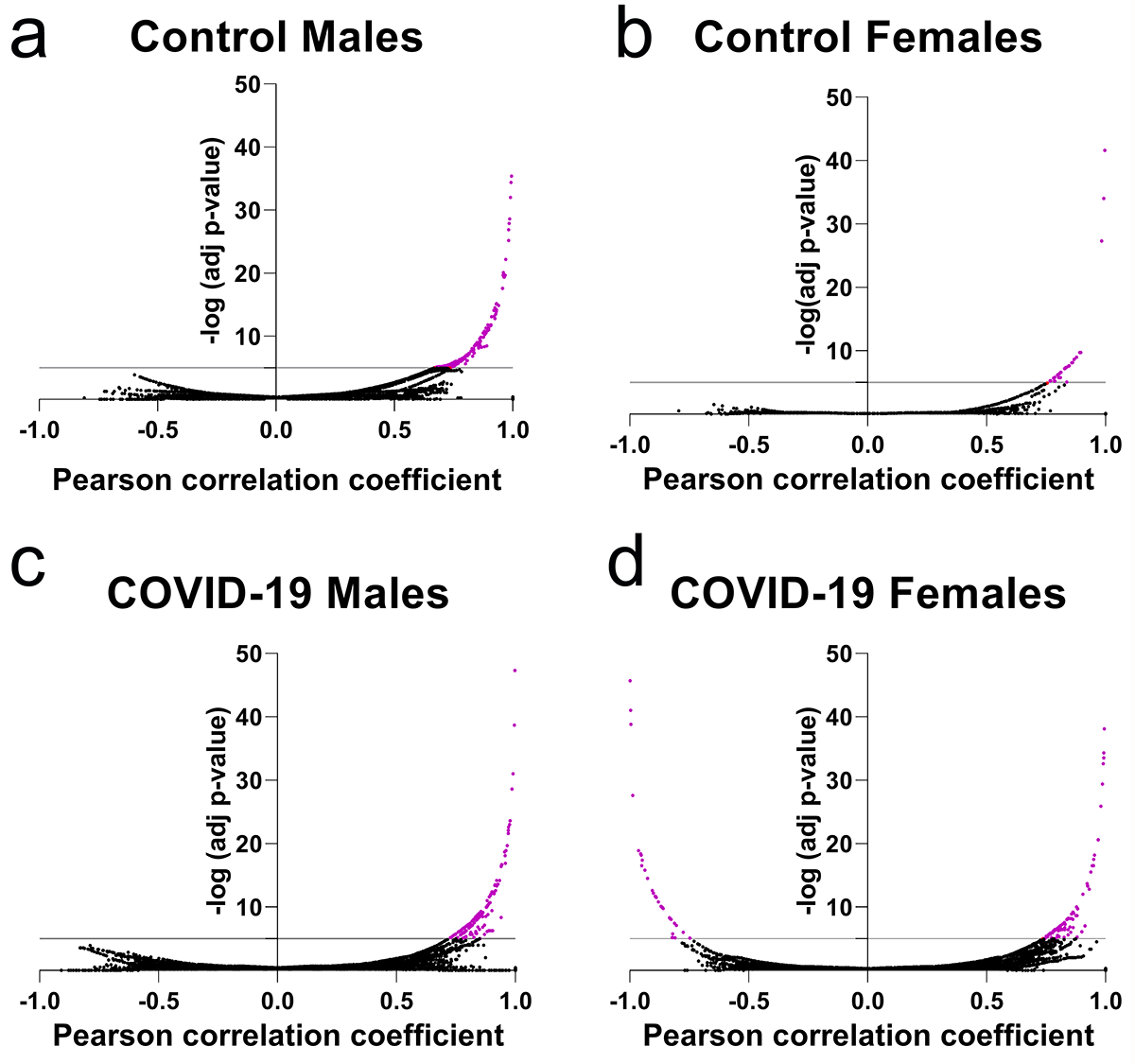

Supplement: Supplementary file 9 — Supp Figure 5 [file 41419_2022_4861_MOESM9_ESM.tif]

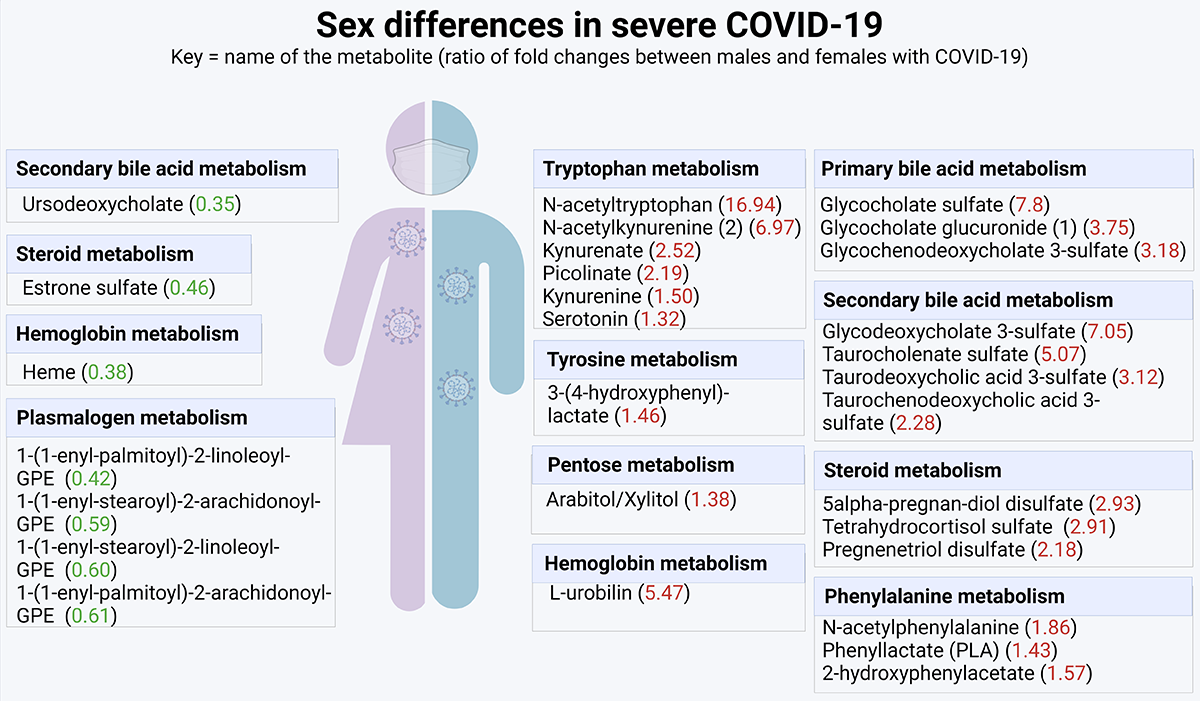

Supplement: Supplementary file 10 — Supp Figure 6 [file 41419_2022_4861_MOESM10_ESM.tif]
