# Supplementary material for: Sex differences in global metabolomic profiles of COVID-19 patients
Source: Cell Death Dis. 2022 May 14;13(5):461. doi: 10.1038/s41419-022-04861-2 (PMC9106988; doi:10.1038/s41419-022-04861-2)
Supplement: Supplementary file 13 — Conformation from all co-authors to agree to add a new co-author. [file 41419_2022_4861_MOESM13_ESM.pdf]

CDDIS-22-0072RR Initial Quality Check

It has come to our attention that your most recent author list differs from the one in your original submission. Please request agreement from all authors including additions and deletions, these can be collected in the following way:

Email your co-authors with the change, and ask them to reply to your email confirming that they agree to these changes. Once you have collected these replies, please combine all of the co-authors' email responses in one document and upload this file to your submission.

Thank you for your email about our manuscript entitled "Sex differences in global metabolomic profiles of COVID-19 patients" (**CDDIS-22-0072RR**). All confirmatory emails are below.

Thank you.

Kind regards,
Andrey

Andrey S. Tsvetkov, Ph.D.
Assistant Professor
Department of Neurology
The University of Texas McGovern Medical School at Houston
6431 Fannin St., MSB 4.142
Houston, Texas 77030
https://med.uth.edu/neurology/specialty-programs/brains-research-laboratory/dr-andrey-tsvetkovs-lab/
Admin Assistant: Nur Compan (Nur.R.CompanZubieta@uth.tmc.edu; 713-500-7545)

**From:**	Diaz Escarcega, Rocio
**To:**	Tsvetkov, Andrey
**Subject:**	Re: our COVID-19 manuscript
**Date:**	Tuesday, April 12, 2022 10:42:33 AM

Hi Andrey,

Yes, I agree.

Best,
Rocio

From: Tsvetkov, Andrey <Andrey.S.Tsvetkov@uth.tmc.edu>
Sent: Tuesday, April 12, 2022 10:26 AM
To: Diaz Escarcega, Rocio <Rocio.DiazEscarcega@uth.tmc.edu>
Subject: our COVID-19 manuscript

Hi Rocio,

In checking in our manuscript submitted to Cell Death & Disease (CDDIS-22-0072RR; Sex differences in global metabolomic profiles of COVID-19 patients), it has come to their attention that the following must be addressed before we can proceed. I should email all co-authors with the addition of **Guadalupe J. Ortiz**, and ask them to reply to my email confirming that they agree to the addition. Once I have collected these replies, I should then combine all of the co-authors' email responses in one document and upload this file to our submission. Please let me know if you are OK with adding Guadalupe J. Ortiz to our manuscript co-authors.

Thank you.
Andrey

From: Honarpisheh, Pedram
To: Tsvetkov, Andrey
Subject: Re: our COVID-19 manuscript
Date: Tuesday, April 12, 2022 1:04:38 PM

I agree with the addition of **Guadalupe J. Ortiz** as a co-author.

Thanks,
Pedram

From: Tsvetkov, Andrey <Andrey.S.Tsvetkov@uth.tmc.edu>
Sent: Tuesday, April 12, 2022 10:26 AM
To: Honarpisheh, Pedram <Pedram.Honarpisheh@uth.tmc.edu>
Subject: our COVID-19 manuscript

Hi Pedram,

In checking in our manuscript submitted to Cell Death & Disease (CDDIS-22-0072RR; Sex differences in global metabolomic profiles of COVID-19 patients), it has come to their attention that the following must be addressed before we can proceed. I should email all co-authors with the addition of **Guadalupe J. Ortiz**, and ask them to reply to my email confirming that they agree to the addition. Once I have collected these replies, I should then combine all of the co-authors’ email responses in one document and upload this file to our submission. Please let me know if you are OK with adding Guadalupe J. Ortiz to our manuscript co-authors.

Thank you.
Andrey

Tsvetkov, Andrey

From: Delevati Colpo, Gabriela
Sent: Tuesday, April 12, 2022 10:48 AM
To: Tsvetkov, Andrey
Subject: RE: our COVID-19 manuscript

Hi Andrey,

I am ok with adding Javier!
Thanks for asking!

Thank you,
Gabi

From: Tsvetkov, Andrey
Sent: Tuesday, April 12, 2022 10:28 AM
To: Delevati Colpo, Gabriela <Gabriela.D.Colpo@uth.tmc.edu>
Subject: our COVID-19 manuscript

Hi Gabi,

In checking in our manuscript submitted to Cell Death & Disease (CDDIS-22-0072RR; Sex differences in global metabolomic profiles of COVID-19 patients), it has come to their attention that the following must be addressed before we can proceed. I should email all co-authors with the addition of **Guadalupe J. Ortiz**, and ask them to reply to my email confirming that they agree to the addition. Once I have collected these replies, I should then combine all of the co-authors' email responses in one document and upload this file to our submission. Please let me know if you are OK with adding Guadalupe J. Ortiz to our manuscript co-authors.

Thank you.
Andrey

From: Ahnstedt, Hilda W
To: Tsvetkov, Andrey
Subject: Re: our COVID-19 manuscript
Date: Tuesday, April 12, 2022 10:32:30 AM

Yes, please add Guadalupe J. Ortiz to the manuscript.

Hilda

Hilda Ahnstedt, PhD

Senior Program Manager

UTHealth Houston | The University of Texas Health Science Center at Houston

McGovern Medical School

Neurology | Grants and Contracts | The Biorepository of Neurological Disorders

6431 Fannin St | MSB 7.100 | Houston, TX 77030

713-500-7742 Phone |802-488-4616 Cell

From: Tsvetkov, Andrey <Andrey.S.Tsvetkov@uth.tmc.edu>
Sent: Tuesday, April 12, 2022 10:28
To: Ahnstedt, Hilda W <Hilda.W.Ahnstedt@uth.tmc.edu>
Subject: our COVID-19 manuscript

Hi Hilda,

In checking in our manuscript submitted to Cell Death & Disease (CDDIS-22-0072RR; Sex differences in global metabolomic profiles of COVID-19 patients), it has come to their attention that the following must be addressed before we can proceed. I should email all co-authors with the addition of **Guadalupe J. Ortiz**, and ask them to reply to my email confirming that they agree to the addition. Once I have collected these replies, I should then combine all of the co-authors’ email responses in one document and upload this file to our submission. Please let me know if you are OK with adding Guadalupe J. Ortiz to our manuscript co-authors.

Thank you.
Andrey

From: Couture, Lucy
To: Tsvetkov, Andrey
Subject: Re: our COVID-19 manuscript
Date: Tuesday, April 12, 2022 10:37:21 AM

I am OK with the addition.

Get Outlook for iOS

From: Tsvetkov, Andrey <Andrey.S.Tsvetkov@uth.tmc.edu>
Sent: Tuesday, April 12, 2022 10:28:39 AM
To: Couture, Lucy <Lucy.Couture@uth.tmc.edu>
Subject: FW: our COVID-19 manuscript

Hi Lucy,

In checking in our manuscript submitted to Cell Death & Disease (CDDIS-22-0072RR; Sex differences in global metabolomic profiles of COVID-19 patients), it has come to their attention that the following must be addressed before we can proceed. I should email all co-authors with the addition of **Guadalupe J. Ortiz**, and ask them to reply to my email confirming that they agree to the addition. Once I have collected these replies, I should then combine all of the co-authors' email responses in one document and upload this file to our submission. Please let me know if you are OK with adding Guadalupe J. Ortiz to our manuscript co-authors.

Thank you.
Andrey

**From:**	S Juneja
**To:**	Tsvetkov, Andrey
**Subject:**	Re: our COVID-19 manuscript
**Date:**	Tuesday, April 12, 2022 6:51:09 PM

****** EXTERNAL EMAIL ******

Good afternoon,

Yes, I am okay with the addition!

Best,
Shivanki Juneja

On Tue, Apr 12, 2022 at 10:29 AM Tsvetkov, Andrey <Andrey.S.Tsvetkov@uth.tmc.edu> wrote:

> Hi Shivanki,
>
> In checking in our manuscript submitted to Cell Death & Disease (CDDIS-22-0072RR; Sex differences in global metabolomic profiles of COVID-19 patients), it has come to their attention that the following must be addressed before we can proceed. I should email all co-authors with the addition of **Guadalupe J. Ortiz**, and ask them to reply to my email confirming that they agree to the addition. Once I have collected these replies, I should then combine all of the co-authors' email responses in one document and upload this file to our submission. Please let me know if you are OK with adding Guadalupe J. Ortiz to our manuscript co-authors.
>
> Thank you.
>
> Andrey

--
Shivanki Juneja
University of Pennsylvania '20
B.A. in Neuroscience

From: Ortiz IV, Guadalupe J
To: Tsvetkov, Andrey
Subject: RE: our COVID-19 manuscript
Date: Wednesday, April 13, 2022 11:17:24 AM

Hi Andrey,

I accept to be added as a co-author to the manuscript titled: "Sex differences in global metabolomic profiles of COVID-19 patients". Please let me know if you need any further information from my end.

With respect,

Javier Ortiz

Javier Ortiz IV, PhD
Research Coordinator II
UTHealth | The University of Texas Health Science Center at Houston
Neurocognitive Disorders Center
Department of Neurology
1941 East Road, Suite 4358
Houston, TX 77054
Phone: 713-486-0505
Email: Guadalupe.J.Ortiz@uth.tmc.edu

From: Tsvetkov, Andrey
Sent: Wednesday, April 13, 2022 10:01 AM
To: Ortiz IV, Guadalupe J <Guadalupe.J.Ortiz@uth.tmc.edu>
Subject: our COVID-19 manuscript

Hi Javier,

Your OK is also needed.

In checking in our manuscript submitted to Cell Death & Disease (CDDIS-22-0072RR; Sex differences in global metabolomic profiles of COVID-19 patients), it has come to their attention that the following must be addressed before we can proceed. I should email all co-authors with the addition of you, **Guadalupe J. Ortiz**, and ask all to reply to my email confirming that they agree to the addition. Once I have collected these replies, I should then combine all of the co-authors' email responses in one document and upload this file to our submission. Please let me know if you are OK with adding Guadalupe J. Ortiz to our manuscript co-authors.

Thank you.
Andrey

**From:**	Torres, Glenda L
**To:**	Tsvetkov, Andrey
**Subject:**	RE: our COVID-19 manuscript
**Date:**	Tuesday, April 12, 2022 10:50:21 AM
**Attachments:**	image002.png

Good morning,
I accept the addition of Guadalupe Ortiz as an author to the manuscript.

Warm Regards,

Glenda Libby Torres, MS

Program Manager-Research

Neurocritical Care

UTHealth The University of Texas Health Science Center at Houston | McGovern Medical School

The Vivian L. Smith Department of Neurosurgery
6431 Fannin St. | MSB 7.154 | Houston, TX 77030
713.500.5638 tel | 713.500.0665 fax |

From: Tsvetkov, Andrey <Andrey.S.Tsvetkov@uth.tmc.edu>
Sent: Tuesday, April 12, 2022 10:46 AM
To: Torres, Glenda L <Glenda.L.Torres@uth.tmc.edu>
Subject: our COVID-19 manuscript

Hi Glenda,

In checking in our manuscript submitted to Cell Death & Disease (CDDIS-22-0072RR; Sex differences in global metabolomic profiles of COVID-19 patients), it has come to their attention that the following must be addressed before we can proceed. I should email all co-authors with the addition of **Guadalupe J. Ortiz**, and ask them to reply to my email confirming that they agree to the addition. Once I have collected these replies, I should then combine all of the co-authors' email responses in one document and upload this file to our submission. Please let me know if you are OK with adding Guadalupe J. Ortiz to our manuscript co-authors.

Thank you.
Andrey

From: James Sollome
To: Tsvetkov, Andrey
Subject: RE: our COVID-19 manuscript
Date: Tuesday, April 12, 2022 11:13:33 AM
Attachments: image002.png
image003.png
image004.png

****** EXTERNAL EMAIL ******

Hi Andrey,

Yes, I am okay with the addition of Guadalupe J. Ortiz as a co-author.

Kind Regards,
James

James Sollome, Ph.D.
Study Director, Discovery & Translational Sciences
Metabolon, Inc.

P: 919.592.7714
jsollome@metabolon.com
617 Davis Drive, Suite 100, Morrisville, NC 27560
P.O. Box 110407, Research Triangle Park, NC 27709
www.metabolon.com

METABOLON

From: Tsvetkov, Andrey <Andrey.S.Tsvetkov@uth.tmc.edu>
Sent: Tuesday, April 12, 2022 11:32 AM
To: James Sollome <jsollome@metabolon.com>
Subject: our COVID-19 manuscript

CAUTION: This email originated from outside of Metabolon. Do not click links or open attachments unless you recognize the sender and know the content is safe.

Hi James,

In checking in our manuscript submitted to Cell Death & Disease (CDDIS-22-0072RR; Sex differences in global metabolomic profiles of COVID-19 patients), it has come to their attention that the following must be addressed before we can proceed. I should email all co-authors with the addition of **Guadalupe J. Ortiz**, and ask them to reply to my email confirming that they agree to the addition. Once I have collected these replies, I should then combine all of the co-authors' email responses in one document and upload this file to our submission. Please let me know if you are OK with adding Guadalupe J. Ortiz to our manuscript co-authors.

Thank you.

From: Natalie Tabor
To: Tsvetkov, Andrey
Subject: Re: FW: our COVID-19 manuscript
Date: Wednesday, April 13, 2022 8:52:25 AM

****** EXTERNAL EMAIL ******

Yes, I am okay with that.

Congrats on finishing it.

Best,

Natalie

On Tue, Apr 12, 2022 at 10:38 AM Tsvetkov, Andrey <Andrey.S.Tsvetkov@uth.tmc.edu> wrote:

> Hi Natalie,
>
> In checking in our manuscript submitted to Cell Death & Disease (CDDIS-22-0072RR; Sex differences in global metabolomic profiles of COVID-19 patients), it has come to their attention that the following must be addressed before we can proceed. I should email all co-authors with the addition of **Guadalupe J. Ortiz**, and ask them to reply to my email confirming that they agree to the addition. Once I have collected these replies, I should then combine all of the co-authors' email responses in one document and upload this file to our submission. Please let me know if you are OK with adding Guadalupe J. Ortiz to our manuscript co-authors.
>
> Thank you.
>
> Andrey

--
Natalie Tabor
Virginia Tech | Class of 2020 | Magna Cum Laude
B.S Human Nutrition, Foods, and Exercise
443-253-0442

Andrey

DISCLAIMER: This email and any file transmitted with it are confidential and intended solely for the use of the individual or entity to whom they are addressed. If you are not the named addressee, you should not disseminate, distribute or copy this e-mail. Please notify the sender or the system manager and delete or destroy this email and any attachment immediately.

**From:**	Ganesh, Bhanu P
**To:**	Tsvetkov, Andrey
**Subject:**	RE: our COVID-19 manuscript
**Date:**	Tuesday, April 12, 2022 11:06:53 AM

OK!

From: Tsvetkov, Andrey
Sent: Tuesday, April 12, 2022 10:34 AM
To: Ganesh, Bhanu P <Bhanu.P.Ganesh@uth.tmc.edu>
Subject: our COVID-19 manuscript

Hi Dr. Ganesh,

In checking in our manuscript submitted to Cell Death & Disease (CDDIS-22-0072RR; Sex differences in global metabolomic profiles of COVID-19 patients), it has come to their attention that the following must be addressed before we can proceed. I should email all co-authors with the addition of **Guadalupe J. Ortiz**, and ask them to reply to my email confirming that they agree to the addition. Once I have collected these replies, I should then combine all of the co-authors' email responses in one document and upload this file to our submission. Please let me know if you are OK with adding Guadalupe J. Ortiz to our manuscript co-authors.

Thank you.
Andrey

**From:**	Choi, HuiMahn A
**To:**	Tsvetkov, Andrey
**Subject:**	Re: our COVID-19 manuscript
**Date:**	Tuesday, April 12, 2022 10:41:33 AM

Ok with the addition. Thanks

On Apr 12, 2022, at 10:34 AM, Tsvetkov, Andrey <Andrey.S.Tsvetkov@uth.tmc.edu> wrote:

Hi Dr. Choi,

In checking in our manuscript submitted to Cell Death & Disease (CDDIS-22-0072RR; Sex differences in global metabolomic profiles of COVID-19 patients), it has come to their attention that the following must be addressed before we can proceed. I should email all co-authors with the addition of **Guadalupe J. Ortiz**, and ask them to reply to my email confirming that they agree to the addition. Once I have collected these replies, I should then combine all of the co-authors' email responses in one document and upload this file to our submission. Please let me know if you are OK with adding Guadalupe J. Ortiz to our manuscript co-authors.

Thank you.
Andrey

**From:**	Liu, Fudong
**To:**	Tsvetkov, Andrey
**Subject:**	RE: our COVID-19 manuscript
**Date:**	Tuesday, April 12, 2022 10:41:00 AM

Sure no problem! Thanks.

From: Tsvetkov, Andrey
Sent: Tuesday, April 12, 2022 10:35 AM
To: Liu, Fudong <Fudong.Liu@uth.tmc.edu>
Subject: FW: our COVID-19 manuscript

Hi Fudong,

In checking in our manuscript submitted to Cell Death & Disease (CDDIS-22-0072RR; Sex differences in global metabolomic profiles of COVID-19 patients), it has come to their attention that the following must be addressed before we can proceed. I should email all co-authors with the addition of **Guadalupe J. Ortiz**, and ask them to reply to my email confirming that they agree to the addition. Once I have collected these replies, I should then combine all of the co-authors' email responses in one document and upload this file to our submission. Please let me know if you are OK with adding Guadalupe J. Ortiz to our manuscript co-authors.

Thank you.
Andrey

From: McCullough, Louise D
To: Tsvetkov, Andrey
Subject: Re: our COVID-19 manuscript
Date: Tuesday, April 12, 2022 8:17:09 PM

You bet!

Sent from my iPhone

> On Apr 12, 2022, at 10:35 AM, Tsvetkov, Andrey <Andrey.S.Tsvetkov@uth.tmc.edu> wrote:
>
> Hi Louise,
>
> In checking in our manuscript submitted to Cell Death & Disease (CDDIS-22-0072RR; Sex differences in global metabolomic profiles of COVID-19 patients), it has come to their attention that the following must be addressed before we can proceed. I should email all co-authors with the addition of **Guadalupe J. Ortiz**, and ask them to reply to my email confirming that they agree to the addition. Once I have collected these replies, I should then combine all of the co-authors' email responses in one document and upload this file to our submission. Please let me know if you are OK with adding Guadalupe J. Ortiz to our manuscript co-authors.
>
> Thank you.
> Andrey
